# The Effect of Direct-Fed Lactobacillus Species on Milk Production and Methane Emissions of Dairy Cows

**DOI:** 10.3390/ani13061018

**Published:** 2023-03-10

**Authors:** S. Richard O. Williams, Joe L. Jacobs, Subhash Chandra, Martin Soust, Victoria M. Russo, Meaghan L. Douglas, Pablo S. Alvarez Hess

**Affiliations:** 1 Agriculture Victoria Research, Ellinbank, VIC 3821, Australia; 2 Centre for Agricultural Innovation, School of Agriculture and Food, Faculty of Veterinary and Agricultural Sciences, The University of Melbourne, Parkville, VIC 3010, Australia; 3 Agriculture Victoria, Tatura, VIC 3616, Australia; 4 Terragen Biotech Pty Ltd., Coolum Beach, QLD 4573, Australia

**Keywords:** direct-fed microbials, methane yield, carbon dioxide, sulfur hexafluoride, feed conversion efficiency, cattle

## Abstract

**Simple Summary:**

Mitigating methane emissions from ruminants requires strategies that are sustainable and acceptable to both consumers and producers. Direct-fed microbials could meet these requirements. Forty Holstein-Friesian cows were randomly allocated one of two treatments, a control or control plus a direct-fed microbial (MYLO^®^—a mixture of *Lactobacillus* species; Terragen Biotech Pty Ltd., Coolum Beach, Queensland, Australia). Adding the direct-fed microbial had no significant effect on feed intake, milk yield, feed conversion efficiency, or methane parameters. While these results are contrary to our expectations, all were numerically in a favorable direction. Given there are reports that diet and dose rate may impact the size of any effect, we recommend a dose–response study be undertaken using a basal diet that is commonly used in pasture-based dairy systems.

**Abstract:**

Using direct-fed microbials to mitigate enteric methane emissions could be sustainable and acceptable to both consumers and producers. Forty lactating, multiparous, Holstein-Friesian cows were randomly allocated one of two treatments: (1) a base of ad libitum vetch (Vicia sativa) hay and 7.0 kg DM/d of a grain mix, or (2) the basal diet plus 10 mL of MYLO^®^ (Terragen Biotech Pty Ltd., Coolum Beach, Queensland, Australia) delivering 4.17 × 10^8^ cfu of *Lactobacillus* per mL. Neither feed intake (25.4 kg/d vs. 24.8 kg/d) nor milk yield (29.9 vs. 30.3 kg/d) were affected by treatment. Feed conversion efficiency was not affected by treatment when expressed on an energy-corrected milk basis (1.15 vs. 1.18 kg/kg DMI). Neither methane yield (31.6 vs. 31.1 g/kg DMI) nor methane intensity (27.1 vs. 25.2 g/kg energy corrected milk) were affected by treatments. While these results are contrary to our expectations and not significant, all were numerically in a favorable direction. Given there are reports that diet and dose rate may impact the size of any effect, we recommend a dose–response study be undertaken using a basal diet that is commonly used in pasture-based dairy systems.

## 1. Introduction

Many dietary strategies to mitigate ruminal methanogenesis have been proposed but few have been effective long term without undesirable effects on animal health or productivity being observed [1]. Using direct-fed microbials as a mitigation strategy is an option that could be sustainable and acceptable to both consumers and producers [2]. Additional benefits of direct-fed microbials in ruminant production are improved animal productivity and health [3]. The most common direct-fed microbials used in ruminant production are *Propionibacterium* and *Lactobacillus* spp. [3]. Originally used in growing ruminants to improve feed utilization, advancements have seen the use of direct-fed microbials expand into mature animals where they are used to improve fiber digestion and reduce the likelihood of ruminal acidosis [3].

Feed conversion efficiency (FCE) has been shown to be improved in beef cattle [4] and growing lambs [5] with the feeding of yeast (*Saccharomyces cerevisiae*). However, there are also reports of no effect (e.g., [6]) when yeast is offered. Feeding bacteria have also been shown to have beneficial effects on FCE, with the feeding of *Lactobacillus* having a positive effect on FCE in beef cattle [7] and increased milk production in primiparous dairy cows [8]. However, there is also a report of *Lactobacillus* having no effect on milk yield or FCE of primiparous dairy cows [2]. Feeding *Propionibacterium* as part of a total mixed ration (TMR) resulted in an increase in milk production and dry matter intake in multiparous cows, but FCE was not reported [9]. Similarly, cows of mixed parity offered *Propionibacterium* produced the same amount of milk but ate less, resulting in net energy usage for milk production being 4.4% lower in the treatment cows [10].

The production of methane in cattle can be inhibited in several ways, one of which is using the hydrogen required for the formation of methane in other metabolic processes. One such process is the production of propionate [11]. Propionate is produced when lactic acid is utilized by *Propionibacterium*, with the growth of *Propionibacterium* encouraged by the presence of *Lactobacillus* that produce lactic acid. Thus, supplementing the rumen with these bacteria should result in a lowering of methane production, and this has been observed in vitro [12,13]. However, the in vivo situation is not as clear. Methane yield (g CH_4_/kg DMI) was not affected when beef heifers were given *Propionibacterium* in a high-grain TMR [14] but was reduced in beef heifers given *Propionibacterium* in a high-forage TMR [15]. Methane yield of dairy cows offered *Propionibacterium* in a TMR was not reported [10], but the observed difference in proportions of volatile fatty acids (VFA) reflected a 12% reduction in methane yield based on Equation (1) ([16], Equation (4)).

MeY = 3.28(A + B)/P + 7.60(1)

where MeY is methane yield, A is acetate, B is butyrate, and P is propionate, all as a proportion of total fatty acids.

While offering *Propionibacterium* to cattle has been shown to reduce the methane yield of animals offered high-forage diets, the in vivo effect of *Lactobacillus* on methane yield of cattle has not been reported previously.

We hypothesized that when compared to cows offered a basal diet, cows offered the basal diet plus a mixture of *Lactobacillus* bacteria would have (1) greater FCE (kg milk/kg DMI), (2) greater milk production, and (3) lower methane yield.

## 2. Materials and Methods

### 2.1. Cows, Diets, and Management

In total, 40 lactating, multiparous, Holstein-Friesian cows producing 35.3 ± 4.00 kg milk/d (mean ± standard deviation) at 4.2 ± 2.04 parities, 78 ± 15.6 days in milk, and with a body weight of 590 ± 47.1 kg were used in the experiment. Each of the 2 treatments were allocated to cows at random (20 cows per treatment), subject to the treatment groups balanced for days in milk, bodyweight, milk yield, and parity using the COVDESIGN procedure in GENSTAT 21 (VSN International, Hemel Hempstead, UK). The two dietary treatments consisted of a base of ad libitum vetch (*Vicia sativa*) hay and 7.0 kg DM/d of a grain mix (rolled barley grain, 294 g/kg DM; rolled corn grain, 280 g/kg DM; rolled wheat grain, 148 g/kg DM; solvent extracted canola meal, 199 g/kg DM; molasses, 15 g/kg DM; sodium bicarbonate, 15 g/kg DM; canola oil 11 g/kg DM; limestone, 10 g/kg DM; and minerals, 28 g/kg DM). The control diet (CON) consisted of the basal diet, while the treatment diet (DFM) consisted of the basal diet plus MYLO^®^ (Terragen Biotech Pty Ltd., Coolum Beach, Queensland, Australia) at the manufacturer’s recommended rate of 10 mL/d. MYLO^®^ contains three species of Lactobacilli; *Lactobacillus buchneri*, *Lactobacillus casei*, and *Lactobacillus paracasei*, with a total *Lactobacillus* concentration of 4.17 × 10^8^ cfu/mL at the time of manufacture, meaning we offered 4.17 × 10^9^ cfu/d. The grain mix was delivered via the feed system in the dairy, then MYLO^®^ was manually placed on top of the grain for those cows on the DFM treatment.

All cows were offered a common diet in the lead up to the experiment. During days 1 to 5 (covariate period) all cows were offered daily the same mix and quantity of grain as already described and perennial ryegrass (*Lolium perenne* L.) pasture at allowance of about 20 kg DM/d to grazing height. On days 6 to 34 of the experiment (adaptation period) all cows were offered their experimental diet. Methane measurements were undertaken on days 35 to 39.

### 2.2. Feed and Milk

The grain mix, and MYLO^®^ as appropriate, were offered to cows in two equal portions daily, one portion at each milking (06:00 h and 14:30 h). Throughout the experiment, hay intakes of individual cows were continuously measured by means of feed bins mounted on load cells that were electronically monitored by linking the bin weight data to electronic identification of individual cows (Gallagher Animal Management Systems, Hamilton, New Zealand), as explained by Moate et al. [17]. The feed bins were located under a small roof to ensure that rain would not compromise feed intake measurements. Between milkings, cows had free access to the electronic feeders and to a loafing area. Water was available ad libitum in the loafing areas.

Refusals of grain mix were collected and weighed, with proportions of each component being assumed to be the same as that offered on a wet basis. Dry matter intake was determined on days 35 to 40. The grain mix offered was representatively sampled on days 35 to 40. Vetch hay was representatively sampled once per day, when the feed bins were filled. Dry matter concentration of offered feeds was determined by drying feed samples in a forced draft oven at 105 °C for 24 h. Additional samples of each feed were kept frozen at −18 °C, bulked within feed over the duration of the experiment, then subsequently oven-dried at 65 °C for 72 h and ground to pass through a 0.5 mm screen. Feed samples were analyzed for nutritive characteristics (Table 1) by wet chemistry [18] at a commercial laboratory (Dairy One Forage Laboratory, Ithaca, NY, USA).

Milk yield was measured for each cow at each milking using a DeLaval milk metering system (MM27BC; DeLaval International, Tumba, Sweden). Milk samples were collected from individual cows during morning and afternoon milkings on days 1 to 4 (covariate period); 8 (transition period); 15, 22, and 29 (adaptation period); and 35 to 39 (measurement period). Fat, protein, and lactose in these milk samples were measured by means of a mid-infrared milk analyzer (Bentley FTS, Bentley Instruments, Chaska, MN, USA). Energy-corrected milk (ECM), standardized to 4.0% fat and 3.3% protein, was calculated using Equation (2) [19].

ECM (kg/cow per d) = milk yield (kg) × (376 × fat% + 209 × protein% + 948)/3138(2)

The liveweight of each cow was measured twice daily by walking the cows over electronic scales (AWS100; DeLaval International AB, Tumba, Sweden) after each milking. The body condition score of each cow was measured automatically twice daily (BCS; DeLaval International AB, Tumba, Sweden).

### 2.3. Methane Emissions

The modified sulfur hexafluoride (SF_6_) tracer technique, as fully described by Deighton et al. [20], was used to estimate methane emissions from individual cows. In this experiment, permeation tubes were filled with about 2.8 g of 99.999% pure SF_6_ (Advanced Specialty Gases, Reno, NV, USA). The release rate of SF_6_ from permeation tubes was determined by incubating the permeation tubes in an oven set at 39 °C and weighing the permeation tubes twice/week for 4 weeks prior to d 29 of the experiment. The SF_6_ release rate from permeation tubes was 5.4 ± 0.45 mg/d (average ± SD) and ranged from 4.4 to 6.4 mg/d. Cows were dosed via the mouth with SF_6_ permeation tubes on d 29. Each permeation tube was encapsulated in a clear gelatin capsule (Size #10 clear veterinary capsule; Torpac Inc., Fairfield, NJ, USA) then administered using a balling gun. From d 35 to 40, evacuated canisters of 800 mL capacity were used to continuously sample eructated gases from a sampling point located just above the cows’ nostrils. An initial sampling rate of ~0.2 mL/min was used to continuously sample eructated gases. Samples of background gases were collected from the right flank of each individual cow into evacuated 800 mL canisters. Canisters were exchanged once per day after the morning milking at ~0700 h for a total of 5 d of collection. Analysis of collected gas samples was carried out the day of sample recovery using gas chromatography [21]. A ‘piston extractor’ (NIWA, Wellington, New Zealand) was used to extract a representative gas sample from the canister and then deliver it to the gas chromatograph [22]; 4 of the 310 gas samples were lost, 1 during analysis and 3 due to failure of the sampling equipment.

### 2.4. Ruminal Fermentation

Ruminal fluid samples (~400 mL) were collected from each cow 4 h after the start of feeding on day 5 and day 40. This 4 h delay from the start of feeding was chosen to coincide with the expected nadir in ruminal pH [23]. An oro-ruminal sampling probe, similar to the one described by Geishauser [24], and a vacuum pump were used to collect samples [25]. 

The pH of the ruminal fluid was immediately measured using a pH meter (FG2, Mettler-Toledo, Schwerzenbach, Switzerland). 

Volatile fatty acid concentrations were analyzed by capillary gas chromatography according to Supelco Bulletin 749D on an Olympus AU400 autoanalyzer after deproteinization with perchloric acid following a procedure based on the method of Erwin et al. [26]. Sample VFA peaks were identified by comparing their retention times with those of a standard mixture of known VFA and quantified using Agilent Chemstations software and Microsoft Excel using 4-methlyvaleric acid as the internal standard. All results were calculated as ppm and converted to mmol/L for subsequent analyses. 

Ammonia-N concentrations were analyzed by the flow injection analysis method based on nitroprusside-salicyate color development chemistry. The analysis relies on the reaction of NH_3_ with salicylate in the presence of free chlorine to form an emerald-green color [27]. Acidified rumen fluid was diluted with 0.5 M HCl and analyzed by Flow Injection Analysis (QuickChem 8500 Series 2 Flow Injection Analyser; Lachat Instruments, Milwaukee, WI, USA) with reference to NH_4_Cl dissolved in 0.1 M HCl standards. 

Protozoa were counted in a 0.5 mL representative sub-sample of ruminal fluid that had been transferred to a 12 mL plastic vial and then diluted with 4.5 mL of a solution containing 4% formalin, 0.9% saline, and 0.4% methylene blue. Counting was done using a Mod–Fuchs–Rosenthal counting chamber and a Leica microscope. Protozoa were differentiated into epidinia (large) and entodiniomorphs [28].

### 2.5. Statistical Analyses

Data in the covariate period and experimental period were expressed as averages for each cow before data analyses. The resulting averages were analyzed by analysis of covariance with days in milk, 7-day mean bodyweight, 7-day mean milk yield, and parity as covariates, as treatments were balanced for these in the design. Measurements of pH, milk yield, milk fat proportion and yield, milk protein proportion and yield, milk lactose proportion and yield, and ECM made just before the experimental period were used as additional covariates for these parameters. 

Distributional assumptions of normality of residuals and constancy of residual variance were checked visually with graphs of residuals plotted against fitted values and histograms and normal quantile plots of residuals. All statistical analyses were performed in Genstat 21 (VSN International, Hemel Hempstead, UK).

## 3. Results

Total feed intake of cows offered the DFM treatment was not different to that of cows offered the CON diet (*p* = 0.333, Table 2). There were no differences in the intake of crude protein (*p* = 0.582), NDF (*p* = 0.602), starch (*p* = 0.561), fat (*p* = 0.515), or metabolizable energy (*p* = 0.553).

Milk yield was not affected by treatment (*p* = 0.553, Table 2), with no treatment differences in ECM (*p* = 0.541) or yields of milk fat (*p* = 0.715), protein (*p* = 0.938), or lactose (*p* = 0.629). Milk composition was also not affected by treatment (*p* > 0.19).

Feed conversion efficiency was not affected by treatment (Table 2) when expressed on a milk-yield basis (*p* = 0.567) or ECM basis (*p* = 0.295).

Body weight gain was not affected by treatment (*p* = 0.164, Table 2), nor was there any effect on the change in body condition score (*p* = 0.746).

Methane emissions were not different between cows in the two treatments (*p* = 0.270, Table 3). Neither methane yield (*p* = 0.749) nor methane intensity (*p* = 0.134) were different between treatments.

The pH values of ruminal fluid samples collected 4 h after the start of feeding were not affected by treatment (*p* = 0.972, Table 4). Treatment also had no effect on ammonia-N (*p* = 0.786) or total VFA concentration (*p* = 0.966). Most individual VFA proportions were not affected by treatment (*p* > 0.13). The exception was iso-butyrate, where the proportion was lower in the DFM cows than in the CON cows (*p* = 0.035). Ratios of VFA were not different between cows in the two treatments (*p* > 0.22). Protozoa counts were also not affected by treatment (*p* > 0.53, Table 4).

## 4. Discussion

Feed conversion efficiency (kg milk/kg DMI) was not affected by treatment, so we reject our first hypothesis. This is unexpected since there are several examples of productivity responses to direct-fed microbials in previous studies. Increases in FCE have been observed when cows were offered *L. acidophilus* and *P. freudenreichii* (rate not reported, [29]), *Propionibacterium* (6 × 10^11^ cfu/d, [10]), and *Propionibacterium* strain P169 (rate not reported, [30]). To our knowledge, there are no previous reports where the same mixture of bacteria as found in MYLO^®^ was fed to dairy cows. The mechanisms behind changes in FCE have been suggested as improved digestion, modified ruminal environmental conditions, or greater microbial protein yield [31]. The case for improved digestion is supported by reports of greater ruminal digestibility of dry matter when dairy cows were supplemented with *E. faecium* at a rate of 5 × 10^9^ cfu/d [32] and greater digestibility of nutrients when offered *L. acidophilus* and *P. freudenreichii* at a rate of 4 × 10^9^ cfu/d [31]. We have not found reports detailing modified ruminal environmental conditions or greater microbial protein yield.

Milk production and ECM yield were not affected by treatment, thus we reject our second hypothesis. No previous reports were found where the same mixture of bacteria as found in MYLO^®^ was fed to dairy cows. However, other combinations of bacteria fed at a similar rate were all reported to increase milk production. For example, compared to cows offered control diets, *E. faecium* offered at a rate of 5 × 10^9^ cfu/d plus yeast resulted in 5.8% greater milk production [32], and *L. acidophilus* and *P. freudenreichii* offered at a rate of 4 × 10^9^ cfu/d resulted in 7.0% more milk and 5.6% more ECM [31]. However, at lower rates of inclusion, the effect has been equivocal. Cows offered *L. plantarum* and *L. casei* at a rate of 1.3 × 10^9^ cfu/d produced 10% more milk than cows on the base diet [8], but supplementing the diet of cows with *L. acidophilus* at 1 × 10^9^ cfu/d and *P. freudenreichii* at 2 × 10^9^ cfu/d resulted in no effect on milk yield [33]. We found only one report where bacteria were offered at a greater rate. When *Propionibacterium* was offered at a rate of 6 × 10^11^ cfu/d, 100 times the dose of *Lactobacillus* in our study, there was no effect on milk yield [10]. These findings indicate the effect of direct-fed microbials on milk yield may be strain dependent and possibly rate dependent.

Methane yield was not affected by treatment; therefore, we reject our third hypothesis. There was also no effect of treatment on methane production or intensity. This contrasts with a previous in vitro study where the addition of *Lactobacillus plantarum* 80 resulted in mitigation of methane by 5 to 15% [12] and a 5 to 15% increase in production of VFA. However, other reports are equivocal and suggest the effect of direct-fed microbials on enteric methane could be dependent on bacterial species, strain, dose, diet, or a combination of all four factors. Screening of direct-fed microbials in vitro for methane mitigating effects showed that only some strains reduced methane compared to the control [34,35]. This effect of strain has been reflected in in vivo studies. When sheep were offered direct-fed microbials at 6 × 10^10^ cfu/d, compared to the control period (no supplementary microbials) there was a 13% reduction in methane yield when *L. pentosus* was used but a 16% increase when *P. freudenreichii* was used [34]. Similarly, compared to dairy cows on a control diet, methane intensity tended to be reduced when *Propionibacterium* P63 at 1 × 10^10^ cfu/d plus *L. rhamnosus* 32 at 1 × 10^10^ cfu/d were offered but there was no effect when *Propionibacterium* P63 plus *L. plantarum* 115 were offered at the same rates [36]. 

A further complication to consider is the possible effect of basal diet on the efficacy of microbial supplements. Philippeau et al. [36] showed that *Propionibacterium* P63 at 1 × 10^10^ cfu/d plus *L. rhamnosus* 32 at 1 × 10^10^ cfu/d tended to decrease methane intensity when cows were fed a low starch diet (2% starch) but had no effect when the same strains were supplemented to cows fed a high starch diet (38% starch). There is one report of *Lactobacillus* having no effect on methane [37], but the *Lactobacillus* were used as a silage inoculant added to the ryegrass at the time of ensiling or to non-inoculated silage 16 h prior to feeding, while we added the *Lactobacillus* at the time of feeding. We were unable to find any reports elucidating the effect of dose of direct-fed microbials on enteric methane. There are thought to be four possible mechanisms by which direct-fed microbials decrease enteric methane production in ruminants: (1) the direct-fed microbials affect the methanogens directly; (2) they affect microbes, such as hydrogen producers, that create the substrates required for methanogenesis; (3) metabolites, such as bacteriocins, of the direct-fed microbials affect the methanogens directly; or (4) the metabolites affect microbes that create the substrates required for methanogenesis [38]. The methane mitigation response appears to be strain dependent and possibly rate and diet dependent. However, there is limited in vivo research available to determine which may have the greater effect on methane production, and it is suggested that further research is required to elucidate the most effective bacterial strains and rates.

The methane yields in our work were greater than expected. Previous work predicts that our cows on the CON diet should have had a methane yield around 21 g/kg DMI [39]. High fiber and low digestibility diets have been reported to result in high methane yield [40,41]. Fiber concentration in the diets of our cows was not high, with NDF only constituting 36% of the feed consumed. However, 85% of the NDF was cellulose and lignin—fiber components generally considered to be of low digestibility. A low digestibility is expected to have resulted in a long retention time, long fermentation duration, high rumination activity, and hence high saliva addition. Low digestibility of our diets is reflected in the low metabolizable-energy density of the hay used and the unexpectedly high pH of our cows 4 h after the start of feeding. The observed high pH is speculated to result from high saliva quantities as part of high rumination activity. The high methane yield of our cows is consistent with their high ruminal pH measured 4 h after the start of feeding [22]. While the methane yield of our cows appears consistent with previous observations of cows on high fiber, low digestibility diets, it is still unclear why our cows had greater than expected methane yield.

Although there was no significant effect of the direct-fed microbials in our experiment, the use of a mixture of *Lactobacillus* to improve production and reduce methane remains plausible. While the numerical differences were small, all metrics moved in the desired direction—FCE was improved (+2.6%) and methane reduced (total, −4.4%; yield, −2.2%; intensity, −7.1%). There are several possible explanations for the small numerical effect observed. *Lactobacillus* has been reported to have the potential to boost ruminal microbes adapting to high lactic acid environments during sub-acute ruminal acidosis [42], but our cows had high ruminal pH of 6.6—an environment where there is no need for adaptation and hence no benefit from the presence of *Lactobacillus*. It is also possible that the dose of *Lactobacillus* used was too low. We offered the *Lactobacillus* at the rate recommended by the manufacturer, but the cows in our experiment ate 20% more than expected, resulting in a lower rate of direct-fed microbial per kg DMI. Alternatively, our method of supplying the direct-fed microbial may be the cause. We offered the DFM only twice per day, during milking, so it is possible that the direct-fed microbial concentration in the rumen declined over time, with any effect only happening for a small portion of the day. However, we consider this to be unlikely since the ruminal pH of our cows (6.6) was in the range conducive to the proliferation of *Lactobacillus* [43]. Finally, it is possible that our adaptation period was too short. A previous study reported that an effect of feeding *Bacillus subtilis* to dairy cows was only observed during weeks 6 to 16 of dosing [44]. This is supported by a brief review of 9 previous reports where most of those studies reporting favorable results from direct-fed microbials dosed animals for 70 days or more. Given our findings and the literature reports, we recommend a dose–response study of more than 5 weeks duration be undertaken using a basal diet that is commonly used in dairy cows.

Ruminal fluid pH, ammonia-N concentration, total VFA concentration, and individual VFA proportions were not affected by treatment. Previous research has supplemented cattle with direct-fed microbials at a lower rate than that in our research and reported no effect of feeding direct-fed microbials on ruminal fermentation parameters. Raeth-Knight et al. [33] reported that supplementing cows with *L. acidophilus* at 1 × 10^9^ cfu/d and *P. freudenreichii* at 2 × 10^9^ cfu/d in a total mixed diet did not affect total VFA concentration, rumen pH, and ammonia-N concentrations in ruminal fluid. The rates of direct-fed microbials used by Raeth-Knight et al. [33] were less than 65% of the *Lactobacillus* dose used in our study. However, supplementing direct-fed microbials at a higher rate has been reported to result in changes to ruminal fermentation. Weiss et al. [10] supplemented lactating dairy cows with *Propionibacterium* strain P169 at 6 × 10^11^ cfu/d, over 140 times the dose of *Lactobacillus* in our study, and found that their treatment cows had lower concentrations of acetate and greater concentrations of propionate and butyrate than their control cows. The absence of differences for VFA concentrations between the DFM treatment and the control in our experiment suggests the DFM treatment did not alter ruminal fermentation pathways. This may have been caused by insufficient metabolically active populations (i.e., low dose rate) in the rumen, which may explain the minor responses in cow performance and enteric methane emissions.

Body weight increased over the course of our experiment, but the gain was not affected by treatment. However, cows offered the DFM diet were numerically heavier at the end of the experiment than those cows offered the CON diet despite both treatments being balanced for liveweight at the start of the experiment. Similarly, dairy cows in the experiment of Nocek and Kautz [32] all lost weight as expected during the first 70 days of lactation but those offered Biomate yeast plus (5 × 10^9^ cfu/d) and 2 *Enterococcos faecium* strains (5 × 10^9^ cfu/d) numerically lost 13 kg less than those on the control diet. Previous studies in beef cattle report mixed effects of direct-fed microbials on body weight. Supplementing the diet of beef steers with *L. acidophilus* at 1 × 10^9^ cfu/d and *P. freudenreichii* at a rate of 1 × 10^6^ cfu/d, a combined rate that was less than 25% of the dose rate used in our study, had no effect on performance [45]. In contrast, Huck et al. [46] reported that *L. acidophilus* at 5 × 10^8^ cfu/d fed for 28 days and then *P. freudenreichii* at 1 × 10^9^ cfu/d fed for 98 days, a combined rate about 30% of ours, resulted in improved daily gain and feed efficiency in beef heifers. These equivocal results support the idea noted above that the effect of direct-fed microbials could be strain, dose, and animal physiology dependent.

## 5. Conclusions

Supplementing the diet of dairy cows with a mixture of three *Lactobacillus* species had no significant effect on FCE (kg milk/kg DMI), milk production, or methane yield of dairy cows in our experiment. However, while the numerical differences were small, all metrics moved in the desired direction. Given our findings and the literature reports, we recommend a dose–response study be undertaken using a basal diet that is commonly used in pasture-based dairy systems.

## Figures and Tables

**Table 1:** Composition of main dietary ingredients (g/kg DM, unless stated otherwise).

Item	Grain Mix	Vetch Hay
Crude protein	131	190
Acid detergent fiber	47.9	389
Neutral detergent fiber	107	438
Lignin	10.2	55.8
Non-fiber carbohydrates	642	203
Starch	511	15.2
Ash	80.7	151
Crude fat	38.6	17.2
Metabolizable energy (MJ/kg DM)	13.4	8.82

**Table 2:** Feed intake and milk yields from cows on each treatment.

Parameter	CON ^1^	DFM	SED ^2^	*p* Value
Feed intake (DMI, kg DM/d)				
Forage	18.4	17.8	0.58	0.303
Grain mix	7.0	7.0	0.05	0.463
Total	25.4	24.8	0.58	0.333
Crude protein	4.47	4.54	0.118	0.582
aNDF	8.94	9.08	0.271	0.602
Starch	3.87	3.87	0.009	0.561
Fat	0.59	0.60	0.011	0.515
Metabolizable energy (MJ/d)	258	262	5.5	0.553
Milk yield (MY, kg/d)	29.9	30.3	0.64	0.553
Energy-corrected milk (ECM)	29.9	30.3	0.62	0.541
Fat	1.2	1.3	0.03	0.715
Protein	0.9	0.9	0.02	0.938
Lactose	1.5	1.5	0.04	0.629
Milk composition (g/kg)				
Fat	41.9	41.4	0.73	0.511
Protein	30.7	30.2	0.39	0.240
Lactose	49.8	49.5	0.26	0.191
FCE (kg MY/kg DMI)	1.16	1.18	0.031	0.567
FCE (kg ECM/kg DMI)	1.15	1.18	0.033	0.295
Body weight gain (kg)	25.8	31.2	3.84	0.164
Condition score change	+0.1	+0.1	0.04	0.746

^1^ CON = control diet, DFM = control diet plus direct-fed microbials. ^2^ SED = standard error of difference.

**Table 3:** Effects of diet on methane emissions, methane yield, and methane intensity.

Parameter	CON ^1^	DFM	SED ^2^	*p* Value
Methane emission (g/d)	799	765	30.8	0.270
Methane yield (g/kg of DMI)	31.6	31.1	1.44	0.749
Methane intensity (g/kg of ECM)	27.1	25.2	1.18	0.134

^1^ CON = control diet, DFM = CON diet plus Lactobacillus. ^2^ SED = standard error of the difference.

**Table 4:** Effect of diet on fermentation parameters in ruminal fluid samples collected 4 h post-feeding on d 35 of the experiment.

Parameter	CON ^1^	DFM	SED ^2^	*p* Value
pH	6.6	6.6	0.06	0.972
NH_3_-N (mg/L)	209	204	18.5	0.786
Total VFA (mg/L)	6173	6154	452	0.966
Individual VFA (mol/100 mol)				
Acetate	60.7	61.2	0.32	0.136
Propionate	16.7	16.4	0.30	0.424
n-Butyrate	15.6	15.6	0.53	0.902
Iso-Butyrate	1.38	1.30	0.036	0.035
n-Valerate	3.10	3.13	0.169	0.876
Iso-Valerate	1.87	1.75	0.077	0.141
Hexanoate	0.62	0.60	0.024	0.341
Heptanoate	0.07	0.07	0.006	0.558
A:P ^3^	3.65	3.73	0.067	0.228
(A + B)/P ^4^	4.68	4.77	0.111	0.437
Protozoa (cells/mL)				
Entodinia	153,326	138,505	23,559	0.535
Epidinia	7974	6908	3509	0.764
Total	155,275	157,888	25,546	0.919

^1^ CON = control diet, DFM = CON diet plus Lactobacillus. ^2^ SED = standard error of the difference. ^3^ Ratio of acetate to propionate. ^4^ Ratio of acetate plus butyrate to propionate.

## Data Availability

All data are available on reasonable request to the corresponding author.
